# Modified paramedian versus conventional paramedian technique in the residency training: an observational study

**DOI:** 10.1186/s12909-020-02118-0

**Published:** 2020-07-02

**Authors:** Shih-Hong Chen, Shiou-Sheng Chen, Chao-Lun Lai, Fang-Ying Su, I-Shiang Tzeng, Li-Kuei Chen

**Affiliations:** 1 Department of Anesthesiology, Taipei Tzu Chi Hospital, Buddhist Tzu Chi Medical Foundation, New Taipei City, Taiwan; 2 Department of Anesthesiology, China Medical University, Taichung City, Taiwan; 3 Department of Anesthesiology, China Medical University Hospital, Taichung City, Taiwan; 4 Department of Urology, School of Medicine, National Yang-Ming University, Taipei, Taiwan; 5 Division of Urology, Taipei City Hospital Heping Fuyou Branch, Taipei, Taiwan; 6 Commission for General Education, National United University, Miaoli City, Taiwan; 7 Department of Internal Medicine, National Taiwan University Hospital Hsin-Chu Branch, Hsin-Chu, Taiwan; 8 Center for Critical Care Medicine, National Taiwan University Hospital Hsin-Chu Branch, Hsin-Chu, Taiwan; 9 Department of Internal Medicine, National Taiwan University College of Medicine, Taipei City, Taiwan; 10 Institute of Epidemiology and Preventive Medicine, College of Public Health, National Taiwan University, Taipei, Taiwan; 11 Biotechology R&D Center, National Taiwan University Hospital Hsin-Chu, Hsin-Chu, Taiwan; 12 Department of Research, Taipei Tzu Chi Hospital, Buddhist Tzu Chi Medical Foundation, New Taipei City, Taiwan

**Keywords:** Patient safety, Paramedian approach, Complication, Residency training

## Abstract

**Background:**

Residency training includes positive and negative aspects. Well-trained doctors must be educated, but the process may bring additional risks to patients. Anesthesiologists’ performance when conducting neuraxial anesthesia is related to their experience. We hypothesized that a modified neuraxial anesthesia method would improve both residency training and patient safety.

**Methods:**

We recruited 518 patients who were scheduled for a cesarean section and used spinal anesthesia (*n* = 256), epidural anesthesia (*n* = 154), and combined spinal–epidural anesthesia (SEA; *n* = 108). We observed and evaluated the anesthesia performance of five second-year resident anesthesiologists in elective cesarean sections using the conventional and modified methods. The number of attempts, implant error rate, and the incidence of complications were recorded and analyzed.

**Results:**

Better success puncture attempts occurred in all three groups when the modified method was applied. For the groups with an implant assessment, the complication rate and implant error rate were lower when using the modified method. We employed generalized estimating equation (GEE) analysis to correct for possible confounding factors. When using the conventional method, the resident anesthesiologists required more attempts, made more implant errors, and caused more complications in patients.

**Conclusions:**

We found that a modified method for neuraxial anesthesia could improve residency performance and patient safety. The modified method may be a suitable training process for resident anesthesiologists when practicing neuraxial anesthesia.

**Trial registration:**

The study was approved by the Research Ethics Committee of National Taiwan University (IRB:200812040R) Clinicaltrials register: NCT03389672.

## Background

Residency training is performed using trial and error. Several studies have shown that the training process, practice period, and resident’s attitude are important factors for determining performance [1–3]. During the training process, neuraxial anesthesia safety is related to the operator’s experience. Ultrasound can improve resident performance [4–7]; however, using ultrasound technologies in well-established training programs may not be practical for all residencies.

Traditional midline and paramedian technique were general practice around the world. Lots of anesthesiologists in Taiwan proceed paramedian approach because paramedian approach bypasses most of the bony structures that may impede the advancement of a needle in the midline approach [8–12]. Previous study showed faster and better success via paramedian approach [9, 13, 14]. However, paramedian approach requires a sharpened three-dimensional insight compared with the midline approach, and needle may be hindered by the barrier when the it passed through the way far away from the midline. We would encounter inferior articular process and pedicle more close to needle pathway, and less inter-lamina foramen diameter in three-dimensional structure especially in obesity patients. We hypothesized that the farther away from the three-dimensional barrier, the better success rate and lower the complications and number of puncture attempts, as compared to a traditional paramedian approach. A modified paramedian approach, which compared to midline approach and traditional approach, was more away from the spinous process, and it may improve residency training and patient safety. The aim of this study was to investigate whether the modified method increased success rate and decreased practice attempts and patient complications.

## Methods

**Fig. 1:**
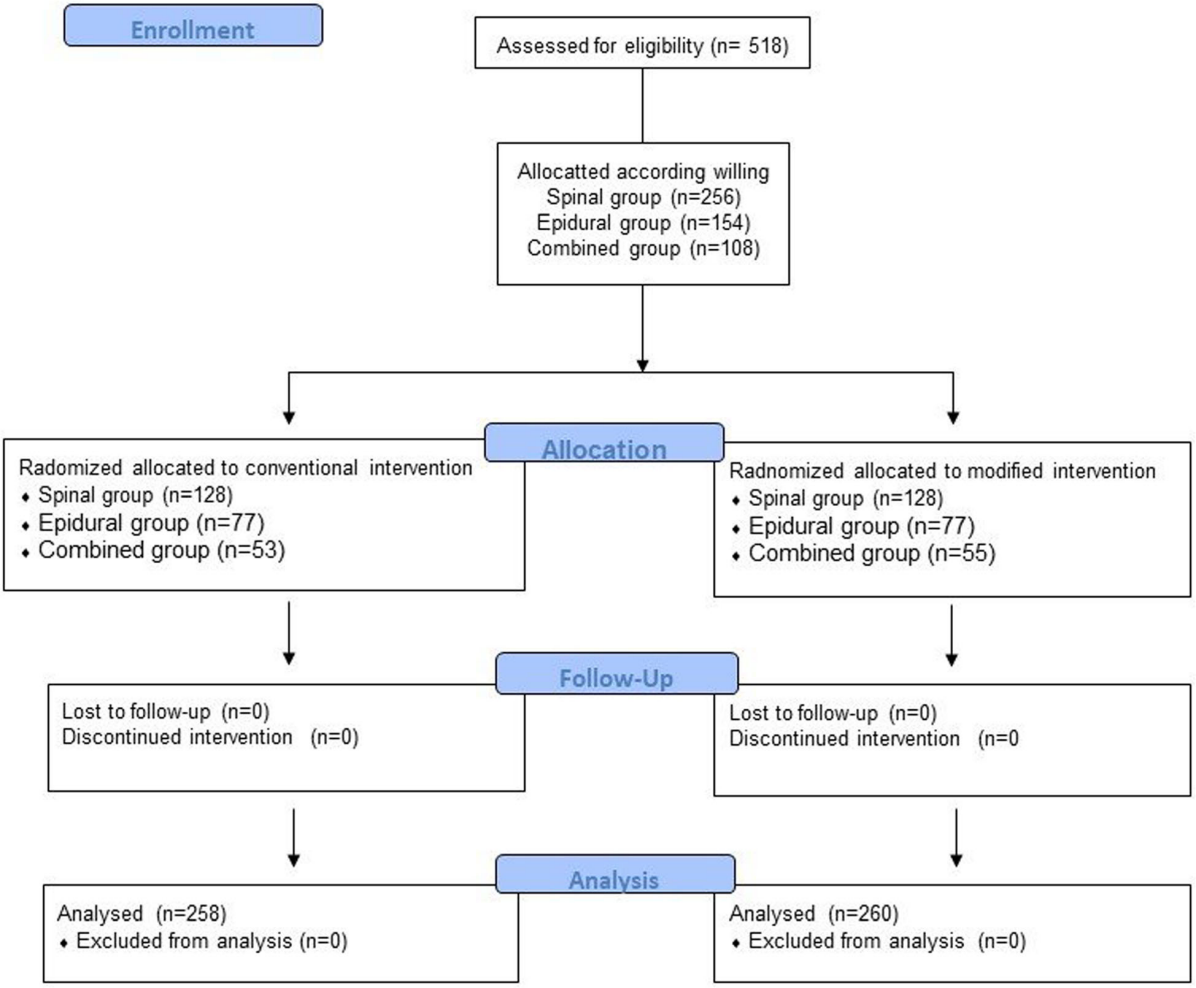
The flow diagram of study

To conduct this prospective study, we observed five resident anesthesiologists practicing regional anesthesia in 518 parturients who received spinal anesthesia (SA), epidural anesthesia (EA), or spinal–epidural anesthesia (SEA) for elective cesarean section from 1 January, 2011, to 30 September, 2012. The study was approved by the Research Ethics Committee of National Taiwan University (IRB:200812040R). All parturients received the information of spinal anesthesia, epidural anesthesia, and SAE anesthesia, and the study protocol. They were assigned to three groups SA, EA, or SEA group by randomized block method and enrolled into the study after they completed the inform consent before the procedure. All parturients in each groups were allocated to conventional method or modified method randomly (Fig. 1). The exclusion criteria were a history of allergy to the medications used in this study, chronic or acute headaches, possible conversion to general anesthesia, and other contraindications to practice (infection, coagulopathy, abnormal spinal anatomy, unstable vital signs, and refusal to participate in the study).

Five second-year resident anesthesiologists had received training for spinal anesthesia, epidural anesthesia, and SEA in their first-year of residency training. The first-year training program included anatomic teaching and clinical performance. Each resident was assigned to performed one of the regional anesthesia via one method without having patients’ information, and the resident would practice the other method next time. All parturients who agreed to join the study were assigned to spinal, Iepidural, and SEA groups according to computer randomized-block number. One study nurse got a sealed-envelop recorded which group the patient was, without knowing other information. The nurse assigned each resident to the operation room to keep the ratio was 1:1 nearly in each group. According to our standard Direct Observation of Procedural Skills assessment and mini-Clinical Evaluation Exercise protocol, one supervisor recorded the measurements for each resident [15].

All parturients received 500 mL of Ringer’s lactate solution intravenously 30 min before anesthesia, after which their vital signs were monitored using equipment including an electrocardiogram, a blood pressure cuff, and an oximeter. Patients were sent to the operation room, and residencies waited in the operation room without knowing the patients’ data before the procedure. In the adopted method, the supervisor was blinded to the patients, surgeons, and anesthesiologists.

All parturients received regional anesthesia in the decubitus position, and the procedures were performed in the L3–4 or L4–5 interspace. The spinal anesthesia was administered using a 27-gauge spinal needle (Becton Dickinson) containing 12 mg of bupivacaine hydrochloride (Marcaine 0.5% Spinal Heavy). The epidural and SEA anesthesias were performed using a 17-gauge Weiss type needle (Becton Dickinson) containing 300 mg of lidocaine hydrochloride (2% Xylocaine injection) without epinephrine. In the SEA group, we performed both a spinal subarachnoid puncture and epidural catheter implant using a combined needle (17-gauge, B. Braun Espocan), and the patients only received an injection of 12 mg (2.2 mL) of bupivacaine hydrochloride into the spinal subarachnoid space. For the conventional method, the injection site was 1 cm lateral and 1 cm caudal to the spinous process, and the needle was directed 45^o^ cephalad and medially to the epidural–subarachnoid space by the operator [16]. For the modified method, the injection site was 0.5 cm lateral, 0.5 cm caudal to the spinous process, perpendicular to skin, and the other process was the same as the conventional method.

### Sample size evaluation

According to our knowledge, there was no previous study, and it was difficult to know adequate sample size. We tried to estimate sample size by using Chi-square test for power calculation. Before the study, our clinical experience has been considered for endpoints and study groups. We calculated the power at the end of study and result showed sufficient sample size (Table 1).

**Table 1:** Power analysis for Chi-square test: alpha = 0.05

	End point	p1 for C	p2 for M	Effect size	Achieved power
Spinal group	Success (attempt = 1)	20.31%	59.38%	0.97	1.000
*N* = 128 per each	Failure	7.81%	1.56%	0.23	0.920
	Complication	1.56%	1.56%	NA	NA
Epidural group	Success (attempt = 1)	14.29%	64.94%	1.45	1.000
*N* = 77 per each	Failure	15.58%	2.60%	0.36	0.965
	Complication	14.29%	1.30%	0.37	0.972
Combined group	Success (attempt = 1)	15.09%	47.27%	0.90	1.000
*N* = 53/55	Failure	22.64%	1.82%	0.50	0.987
	Complication	20.75%	3.64%	0.42	0.963

### Clinical measurement and outcome

The primary outcome of our study was the success rate of attempt with traditional paramedian or modified paramedian technique in all three groups. The secondary outcome included complications in all three groups and implant error in EA and SEA groups.

The number of attempts equated to the number of skin-to-site needle punctures before a successful needle puncture was achieved. Failure (represented by the failure rate) was determined when the number of attempts exceeded four. The success procedure was defined as regional anesthesia was completed at the first-attempt. The implant error rate was defined when a dura puncture occurred, when there was difficulty threading the catheter, or when intravascular catheterization or intrathecal catheterization occurred. Patient outcomes were recorded as all types of complications, including a post dura-puncture headache epidural hematoma, infection, or any unexpected neurologic injury.

### Statistical analysis

Clinical data are expressed as number and percentage (n, %) for categorical variables, or mean ± standard deviation for continuous variables. We compared the demographic and clinical variables of the conventional method and the modified method by using the 2-sample independent t test for continuous variable, and the chi-square test for categoric variable. Because each physician performed the procedure on several study subjects as per our study design, we used generalized estimating equation (GEE) analysis with an independent working correlation matrix to address any correlation problems cause by the clustering of patients for the same operating physician [17–19], and odds ratio were presented to note the significance outcome. A 2-tailed value of *p* < 0.05 was considered significant. SAS statistical software (SAS System for Windows, version 9.4; SAS Institute, Cary, NC, USA) and SPSS version 22 (SPSS Inc., Chicago, Illinois, USA) were used in this study.

## Results

Five second-year resident anesthesiologists performed 256 spinal anesthesias, 154 epidural anesthesias, and 108 SEAs during the study. Patient characteristics are shown in Table 2. There was no statistical difference in age, height, weight, and number of nulliparous or multiparous patients.

**Table 2:** Patient demographic and clinical characteristics

	Conventional Method	Modified Method	*p* value*
Spinal Group (n)	N = 128	N = 128	
Age (years)	30.17 ± 2.53	30.05 ± 2.61	0.697
Height (cm)	159.22 ± 2.78	159.69 ± 2.86	0.185
Weight (kg)	63.98 ± 4.13	64.23 ± 4.27	0.634
BMI (kg/m^2^)	25.23 ± 1.38	25.18 ± 1.36	0.754
Parity, n (%)			0.617
Nulliparous	62 (48.44)	66 (51.56)	
Multiparous	66 (51.56)	62 (48.44)	
Epidural group (n)	N = 77	N = 77	
Age (years)	30.14 ± 2.22	29.92 ± 2.38	0.553
Height (cm)	159.86 ± 2.71	160.04 ± 2.84	0.685
Weight (kg)	64.34 ± 3.78	64.39 ± 4.18	0.936
BMI (kg/m2)	25.17 ± 1.19	25.13 ± 1.30	0.844
Parity, n (%)			0.629
Nulliparous	39 (50.65)	36 (46.75)	
Multiparous	38 (49.35)	41 (53.25)	
Combined group(n)	N = 53	*N* = 55	
Age (years)	30.02 ± 2.26	30 ± 2	0.800
Height (cm)	159.64 ± 2.90	160 ± 3	0.248
Weight (kg)	64 ± 4	64 ± 4	0.401
BMI (kg/m2)	25 ± 1.2	25 ± 1.1	0.908
Parity, n (%)			0.847
Nulliparous	27 (50.94)	27 (49.09)	
Multiparous	26 (49.06)	28 (50.91)	

Values are the mean ± standard deviation or number (percentage)

*Differences between groups were evaluated by the two sample t-test or χ^2^ test

Abbreviation: *BMI* body mass index

*Note that the minimum sample size for conventional and modified method groups were defined without patients’ information so that an estimated prevalence of 50% for modified method compared to conventional method

Across all three groups, using the modified method resulted in fewer attempts than using the conventional method, and residents had the highest number of successful first attempt (modified vs conventional, 59.38% vs 20.31% in spinal group, 64.94% vs 14.29% in epidural group, and 47.27% vs 15.09% in SEA group, *p* < 0.001), indicating that the success rate was higher for the modified method than the other anesthesia methods. The implant error rate in the modified method group showed a higher none-error rate in the epidural (modified vs conventional, 85.71% vs 51.95%, *p* < 0.001) and SEA groups (modified vs conventional, 87.27% vs 35.85%, *p* < 0.001). It showed that junior residents would make less iatrogenic injury via modified method. In the spinal anesthesia group, the complication rate was low using both methods with no statistical difference, whereas a significantly lower complication rate was noted in the modified method (1.3% in epidural group, *p* = 0.001, 3.64% in SEA group, *p* = 0.006) for the epidural anesthesia and SEA groups Complication is related to attempt and implant error, and our complication rate are compatible with other result (Table 3).

**Table 3:** Residency performance outcome and complication

	Conventional Method	Modified Method	*p* value*
Spinal Group			
Attempt Number			< 0.001
1	26 (20.31%)	76 (59.38%)	
2–3	68 (53.13%)	47 (36.72%)	
> 3	24 (18.75%)	3 (2.34%)	
Failure	10 (7.81%)	2 (1.56%)	0.018
Complications ^a^	2 (1.56%)	2 (1.56%)	1.000
Epidural group			
Attempt Number			< 0.001
1	11 (14.29%)	50 (64.94%)	
2–3	24 (31.17%)	19 (24.68%)	
> 3	30 (38.96%)	6 (7.79%)	
Failure	12 (15.58%)	2 (2.60%)	
None error	40 (51.95%)	66 (85.71%)	< 0.001
Implant error ^b^			< 0.001
D	12 (15.58%)	5 (6.49%)	
P	18 (23.38%)	5 (6.49%)	
IV	7 (9.09%)	1 (1.30%)	
Complications ^a^	11 (14.29%)	1 (1.30%)	0.003
Combined group			
Attempt Number			< 0.001
1	8 (15.09%)	26 (47.27%)	
2–3	15 (28.30%)	22 (40.00%)	
> 3	18 (33.96%)	6 (10.91%)	
Failure	12 (22.64%)	1 (1.82%)	
None error	19 (35.85%)	48 (87.27%)	< 0.001
Implant error ^b^			< 0.001
D	10 (18.87%)	2 (3.64%)	
P	19 (35.85%)	4 (7.27%)	
IV	5 (9.43%)	1 (1.82%)	
Complications ^a^	11 (20.75%)	2 (3.64%)	0.006

Values are expressed as number (n(%))

^a^ Complications include infection, epidural hematoma, and post-dural puncture headache

^b^ Implant error included dura puncture, difficulty of catheter threading, blood withdraw, and intrathecal catheterization

Abbreviations: *D* difficulty at threading; *P* dura puncture, *IV* intravascular catheterization

To adjust for confounding factors such as operator, age, and parity, we applied GEE analysis to the results, which was adjusted for the operator, patient’s age, body mass index, parity, and interaction between age and body mass index. The results from the conventional method and the modified method are presented in Table 4. In all three groups, residents had higher Odd ratio for more than one attempt (5.763, 13.739, and 5.354 in spinal, epidural, and SEA group, respectively, *p* < 0.001). The higher Odd ratio of more than one attempt, implant error and complication indicated that junior residents might make patients suffer from more injury via conventional method.

**Table 4:** Association between conventional method versus modified method and clinical outcomes with controlling the factors by GEE

	OR(95%CI)	*p* value
Spinal Group		
Attempt Number		
1	1	
2–4 and failure	5.763 (4.911–6.763)	< 0.001
Complications		
None	1	
Yes	0.848 (0.190–3.783)	0.829
**Epidural group**		
Attempt Number		
1	1	
2–4 and failure	13.739 (11.298–16.709)	< 0.001
Implant error		
None	1	
D	5.766 (3.693–9.002)	< 0.001
P	6.906 (4.482–10.639)	< 0.001
IV	15.251 (3.618–64.298)	< 0.001
Complications		
None	1	
Yes	13.663 (2.143–87.098)	0.006
**Combined group**		
Attempt Number		
1	1	
2–4 and failure	5.354 (3.973–7.214)	< 0.001
Implant error		
None	1	
D	12.744 (3.578–45.392)	< 0.001
P	11.616 (7.549–17.873)	< 0.001
IV	12.801 (3.944–41.547)	< 0.001
Complications		
None	1	
Yes	7.131 (1.804–28.187)	0.005

Abbreviations: *OR* Odd ratio; *D* difficulty at threading; *P* dura puncture, *IV* intravascular catheterization

## Discussion

In our study, the resident anesthesiologists had a higher success rate and lower complication rate when they used the modified paramedian method for spinal anesthesia, epidural anesthesia, and SEA. Resident anesthesiologists need practice and the ability to tolerate failure. Our modified paramedian method lowers the failure rate and accelerates resident competency [2]. One reason for this could be that the shorter distance between the needle puncture site and palpable interspinous space and the lower dimensional barrier significantly improve accuracy.

**Fig. 2:**
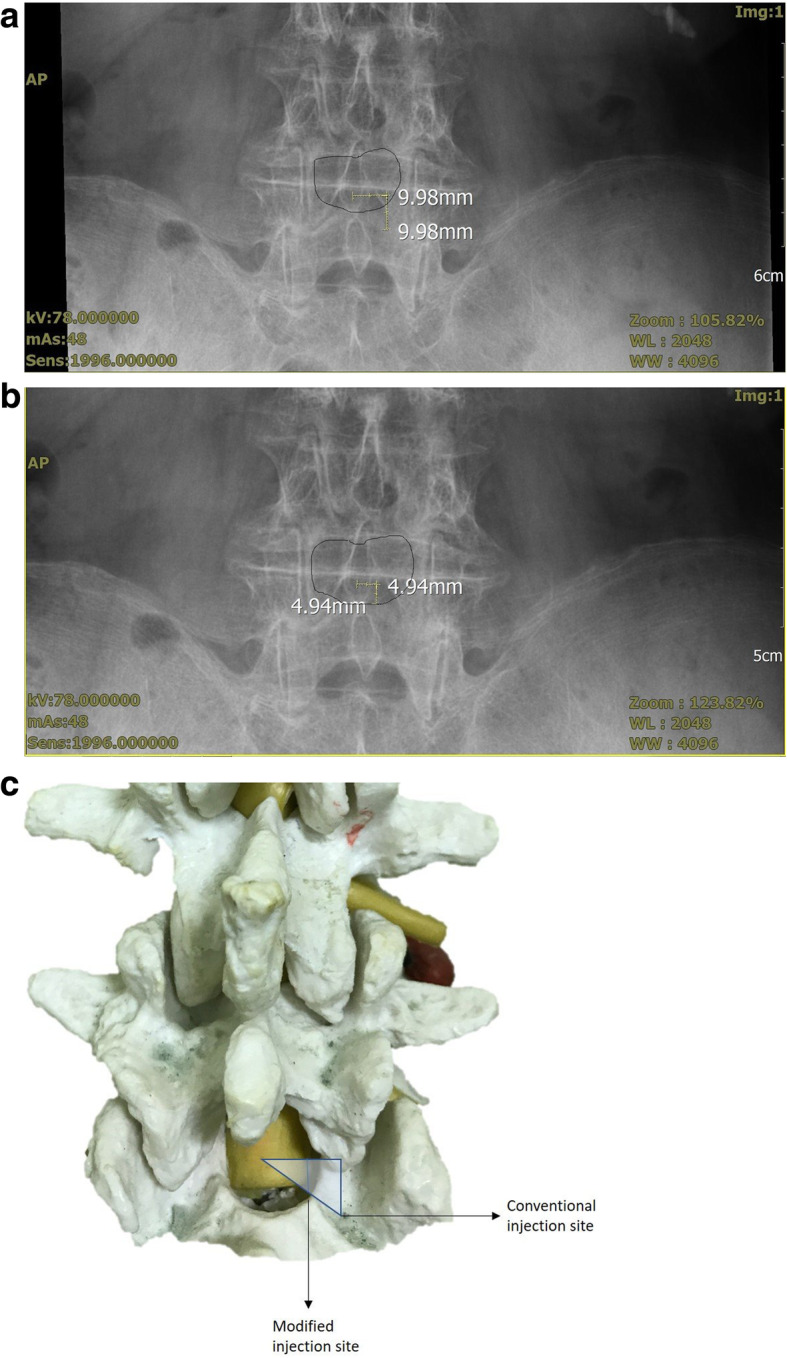
Less three dimension obstacles in modified method (**b**) than in modified method (**a**). **c** It illustrated the differences of modified and conventional injection site

For the successful administration of neuraxial anesthesia, the primary goal is to direct the needle to the interlaminar space and avoid 3-dimensional obstacles such as the spinous process and lamina. With traditional paramedian method, the needle is directed 45o upward to the skin and directed to the midline of spinous process. The horizontal space is usually smaller between interspinous space than interlaminar space, and the spinous process might impede the needle when the insertion site wasn’t away from the midline. The modified technique might increase the probability of needle over the lamina ridge and entered the interlaminar space more easily (Fig. 2a, b, c). Cousins and Bromge described the different paramedian methods used to facilitate the needle walk over the superior ridge of the lamina into the interlaminar space [10]. The shorter skin-to-site distance enhanced the residents’ awareness of the position of the needle tip and helped them to avoid contacting bone structures.

The paramedian approach might be more suitable than the midline approach in various aspects such as fewer dura punctures [11], a lower incidence of epidural catheter errors [8, 20], and an easier approach to continuous spinal anesthesia for older adults [21]. However, previous studies have reported that junior anesthesiologists required fewer attempts or no perceivable difference when they adopted the midline approach [22, 23]. In addition, the paramedian approach requires a larger needle insertion depth and a greater needle puncture angle than the midline approach, and the paramedian vertical measurement gradually diminishes at the L3–5 levels [10]. This approach may be more difficult for junior residents to perform. The modified method, however, was closer to the spinous process than the conventional method, which reserved the benefit of both the midline approach and the paramedian approach.

Our study had several limitations. For example, it was an observational study, and the residents were not assigned the procedure randomly. Second, we didn’t perform the statistic test between independent groups. Third, we couldn’t find previous study, and we didn’t calculate sample size in advance. At the end of study, we calculate the power and the sample size was sufficient. Fourth, it was not a multicenter study and statistical bias may exist in different area. In addition, we did not record the learning summation for the final technical maturation. Due to GEE and GLM have the same coefficients under minor correlation between groups in this study, we finally presented results of analysis using GLM. To our knowledge, it’s not discouraging clinical finding from this study.

## Conclusion

Our modified method improves resident performance and causes fewer patient complications. To improve both residency training and patient safety, we recommend that the modified method to the paramedian approach should be adopted in clinical practice.

## Data Availability

The datasets used and/or analyzed during the current study are available from the corresponding author on reasonable request.

## Abbreviations

SEA: Combined spinal epidural anesthesia
SA: Spinal anesthesia
EA: Epidural anesthesia
